# Improving real‐world myeloma patient access to whole body MRI through “open‐access” knowledge sharing: The UK experience

**DOI:** 10.1002/jha2.25

**Published:** 2020-06-16

**Authors:** Martin F Kaiser, Kevin Boyd, Dow‐Mu Koh, Mihaela Rata, Matthew Blackledge, Christina Messiou

**Affiliations:** ^1^ Division of Molecular Pathology The Institute of Cancer Research London UK; ^2^ Department of Haematology The Royal Marsden Hospital London UK; ^3^ Department of Radiology Royal Marsden Hospital and Institute of Cancer Research London UK; ^4^ Joint Department of Physics The Institute of Cancer Research London UK

In 2016, NICE guidelines [1] positioned whole body‐MRI (WB‐MRI) as the imaging modality of choice for all patients with a suspected diagnosis of myeloma in the UK. Despite these guidelines, a 2017 survey of UK myeloma treatment centers revealed that skeletal survey was the most commonly used imaging for patients with suspected myeloma followed by CT, MRI spine, and then WB‐MRI with no centers reporting use of FDG PET/CT in this setting. One of the identified challenges to providing a WB‐MRI service in the UK was radiologists training [2]. In this letter, we highlight standardization of WB‐MRI and opportunities for training that have resulted in improved access to WB‐MRI for patients with myeloma in clinical practice and clinical trials.

Bone marrow disease imaging by MRI is clinically relevant for management of multiple myeloma patients as per international diagnostic guidelines. A positive MRI defined by the presence of >1 focal lesion >5 mm, is now considered as a high‐risk biomarker stratifying patients for treatment prior to significant cortical bone damage [3]. The radiology department at our institution has actively developed WB‐MRI protocols since 2007 and through successful clinical research achieved implementation as a routine clinical service as early as 2011. The combination of anatomical and functional information delivered by non‐invasive WB‐MRI offers superior sensitivity compared with other imaging techniques in addition to wide anatomical coverage, quantitative response assessments and evaluation of mechanical complications [4]. Although FDG PET/CT also offers both functional and anatomical detail with significant progress toward harmonizing interpretation [5], comparisons with contemporary WB‐MRI protocols have shown superior sensitivity for WB‐MRI [6, 7], on top of its non‐ionizing advantage. Furthermore, the 2016 assessment by NICE revealed a negative net monetary benefit for FDG PET/CT compared to a positive benefit for WB‐MRI. Building on our and other teams’ experience, we sought to standardize acquisition and reporting of WB‐MRI for patients with myeloma, which was published as an international consensus recommendation (MY‐RADS) in 2019 and presented at the Radiological Society of North America in 2018 and 2019 [8]. Specifically, these contemporary protocols incorporate quantitative diffusion weighted and Dixon MRI. We have already demonstrated that MY‐RADS can be successfully applied in a prospective multi‐center clinical trial setting within the IMAGIMM trial (substudy of the MUKnine trial; NCT03188172) across 10 sites thus far and three MRI vendor systems at 1.5T and 3.0T. In parallel to our research, which continues to demonstrate the advantages of WB‐MRI, we have responded to the need for structured education and pro‐active knowledge sharing of protocols for scanning and reporting as an important step for achieving wider access for myeloma patients. All Royal Marsden WB‐MRI protocols are freely available through open access websites (https://www.siemens-healthineers.com/magnetic-resonance-imaging/magnetom-world/clinical-corner/protocols/whole-body-mri/wb-mri-aera-skyra-avanto). We set up and ran educational courses including lectures and interactive workshops hosted by our institution, working closely with educational radiology organizations. In addition, we regularly host visitors to the MRI department to learn first‐hand how we perform and report scans.

We report here on the large number of geographically widely distributed UK radiology units from myeloma treatment centers that have been reached through these initiatives. Since 2017, we have organized four interactive WB‐MRI courses: Royal Marsden Cancer Imaging Perspectives; two courses with the British Institute of Radiology and Prof Hall‐Craggs; one course with the International Cancer Imaging Society. In total, 226 radiologists attended one or more of these interactive courses, many more attending lecture‐based teaching initiatives. In addition, 29 radiologists have been hosted by The Royal Marsden to gain reporting experience. The Royal Marsden Cancer Imaging Perspectives course was the first to incorporate hands on training for radiographers and further practical training sessions have also been successful. Participants were from a wide range of hospitals across the UK, leading to substantive geographical reach of our educational initiative (Figure 1).

**FIGURE 1:**
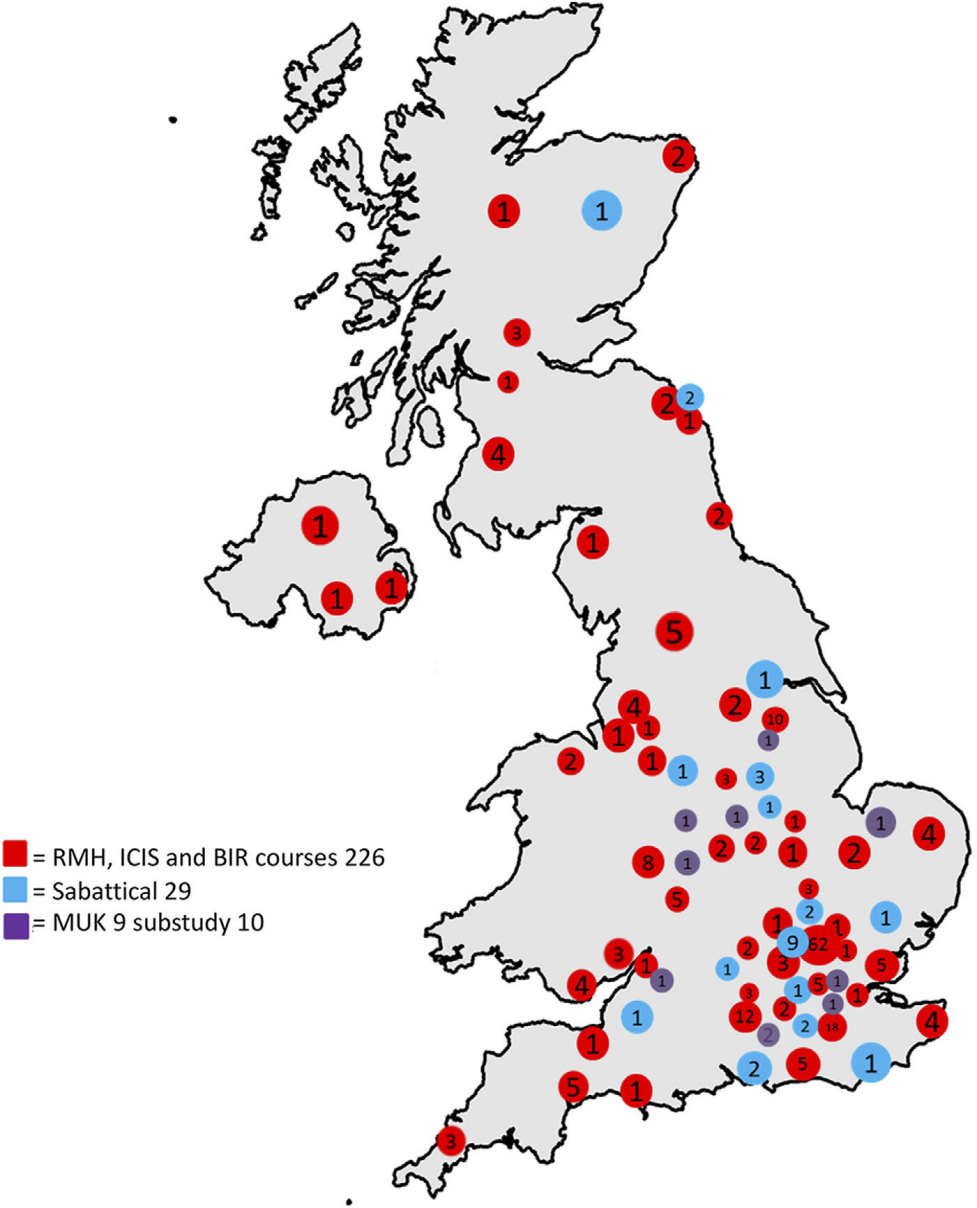
Number and workplace geography of attendees of WB‐MRI educational courses hosted by our institution between 2017 and now, including clinical trial related education/training

While we are cognizant that significant barriers to WB‐MRI access for myeloma patients remain to be overcome, we are highly encouraged by scale of knowledge dissemination through our knowledge sharing strategy. Furthermore, our own survey of course attendees has indicated that although 52% of radiologists were offering a WB‐MRI service in 2017, this rose to 64% after our training. Note that 100% of radiologists believed WB‐MRI to be of clinical benefit and many radiologists anecdotally reported that NICE guidance was used to leverage a successful business case. The commonest reason for not providing the service was lack of MRI capacity (30%). Comparing with data from the Organisation of Economic Cooperation and Development (OECD), the UK has one of the lowest number of MRI systems per million population [9]. Although in part, this will be addressed by the UK governments commitment of £200 million for cancer screening that will include investment in new scanners, creative solutions to overcome staff and MRI scanner shortages are required. Artificial intelligence solutions for rapid image acquisition and automation of lesion detection and quantification are underway (NCT03574454). Increased availability of WB‐MRI will secure benefits for patients that reach far beyond first diagnosis of myeloma with emerging applications for response assessment and surveillance in high risk or asecretory patients [4]. Evidence for use of WB‐MRI in imaging metastatic bone disease [10], and screening of high‐risk populations [11], is also gaining momentum. We believe that knowledge sharing supports patient access to standardized scanning as well as future evidence generation by enabling WB‐MRI clinical trials in the NHS and elsewhere.

## AUTHOR CONTRIBUTIONS

MK and CM designed and wrote manuscript; all authors contributed to stated educational programs and editing the manuscript. Funding: Martin Kaiser was supported through a Jacquelin Forbes‐Nixon Fellowship.

## CONFLICT OF INTEREST

MFK: Abbvie ‐ consultancy; Bristol‐Myers Squibb – consultancy, travel support; Chugai – consultancy; Janssen – consultancy, honoraria; Amgen – consultancy, honoraria; Takeda – consultancy, travel support; Celgene Corporation – consultancy, honoraria, research funding. KDB: Janssen – consultancy, honoraria, travel support; Amgen – honoraria; Takeda – consultancy, honoraria, travel support; Celgene – consultancy, honoraria, travel support; Novartis – consultancy.

## Data Availability

Data sharing is not applicable to this article as no new data were created or analyzed in this study.
